# Stress Assessment by Prefrontal Relative Gamma

**DOI:** 10.3389/fncom.2016.00101

**Published:** 2016-09-22

**Authors:** Jesus Minguillon, Miguel A. Lopez-Gordo, Francisco Pelayo

**Affiliations:** ^1^Department of Computer Architecture and Technology, University of GranadaGranada, Spain; ^2^Research Centre for Information and Communications Technologies, University of GranadaGranada, Spain; ^3^Department of Signal Theory, Telematics and Communications, University of GranadaGranada, Spain; ^4^Nicolo AssociationGranada, Spain

**Keywords:** stress, EEG, ECG, prefrontal relative gamma, heart rate

## Abstract

Stress assessment has been under study in the last years. Both biochemical and physiological markers have been used to measure stress level. In neuroscience, several studies have related modification of stress level to brain activity changes in limbic system and frontal regions, by using non-invasive techniques such as functional magnetic resonance imaging (fMRI) and electroencephalography (EEG). In particular, previous studies suggested that the exhibition or inhibition of certain brain rhythms in frontal cortical areas indicates stress. However, there is no established marker to measure stress level by EEG. In this work, we aimed to prove the usefulness of the prefrontal relative gamma power (RG) for stress assessment. We conducted a study based on stress and relaxation periods. Six healthy subjects performed the Montreal Imaging Stress Task (MIST) followed by a stay within a relaxation room while EEG and electrocardiographic signals were recorded. Our results showed that the prefrontal RG correlated with the expected stress level and with the heart rate (HR; 0.8). In addition, the difference in prefrontal RG between time periods of different stress level was statistically significant (*p* < 0.01). Moreover, the RG was more discriminative between stress levels than alpha asymmetry, theta, alpha, beta, and gamma power in prefrontal cortex. We propose the prefrontal RG as a marker for stress assessment. Compared with other established markers such as the HR or the cortisol, it has higher temporal resolution. Additionally, it needs few electrodes located at non-hairy head positions, thus facilitating the use of non-invasive dry wearable real-time devices for ubiquitous assessment of stress.

## Introduction

According to the definition provided by the American Institute of Stress (AIS), stress in daily-life context is commonly defined as a physical, mental, or emotional strain (for detailed information, please visit the website of the AIS^1^). However, there is no universally accepted definition of stress. Statistics of 2014 in the United States (US) revealed that 77 and 73% US people regularly experience, respectively, physical (e.g., fatigue, headache, and muscle tension), and psychological (e.g., anger, nervous feeling, and lack of energy) symptoms caused by stress. Stress is usually caused by a variety of cognitive, social or physical factors such as job pressure, economic status, health, and relationships. Depending on the positive or negative connotations of stress, this can be classified as eustress (i.e., good stress, e.g., concentration on a task, success, and happiness) or distress (i.e., bad stress, e.g., failure and problems). Regarding the stimulus and response, stress can be acute or chronic. Acute stress is characterized by *fight or flight* responses to unexpected stimuli. Psychological and physiological defense mechanisms are activated and take several minutes to return to relax. Furthermore, chronic stress is caused by daily-life circumstances and can affect the health (e.g., metabolism and immune system).

Regarding the research on stress, this has been under study from several years ago (Selye, 1975a,b; Pearlin et al., 1981; Kingston and Hoffman-Goetz, 1996) to nowadays (Caspi et al., 2003; Aschbacher et al., 2013; Friedman et al., 2014; Mahar et al., 2014; Slavish et al., 2015). It is common to make use of methods to induce stress in subjects in stress-related works. Several methods have been proved to successfully achieve this goal such as the Montreal Imaging Stress Task (MIST; Dedovic et al., 2005), the Trier Social Stress Test (TSST; Kirschbaum et al., 1993), and the Mannheim Multicomponent Stress Test (MMST; Kolotylova et al., 2010). In order to assess stress, various biochemical (e.g., cortisol and salivary alpha-amylase) and physiological (e.g., heart rate, blood pressure, galvanic skin response, and pupil size) markers have been proposed (Schleifer and Okogbaa, 1990; Sayette, 1993; Chandiramani et al., 2007; Ranganathan et al., 2012; Reinhardt et al., 2012; Aschbacher et al., 2013; Michels et al., 2013; Regula et al., 2014; Dimitriev and Saperova, 2015; Slavish et al., 2015; Zschucke et al., 2015). See Bali and Jaggi (2015) for a recent review in methods and assessment in stress studies. Unfortunately, most of the established markers such as the cortisol or the heart rate (HR) cannot be easily implemented on wearable real-time devices for ubiquitous assessment of stress. On the contrary, some neurological markers have better temporal resolution, and therefore they can be implemented on those systems.

Brain activity has been studied under stressful circumstances using, for instance, functional magnetic resonance imaging (fMRI; Dagher et al., 2009; Dedovic et al., 2009b), near-infrared spectroscopy (NIRS; Tanida et al., 2007), positron emission tomography (PET; Nagano-Saito et al., 2013), and electroencephalography (EEG; Seo and Lee, 2010; Brouwer et al., 2011; Papousek et al., 2014). These works demonstrated that stress causes changes in regions of prefrontal and frontal areas such as the orbitofrontal regions, frontal lobes, and medium prefrontal cortex. See Dedovic et al. (2009a) for a review in neuroimaging-based stress studies. Regarding the EEG-based studies, they have suggested that the exhibition or inhibition of certain brain rhythms (e.g., alpha, theta, gamma) in frontal cortical areas reflects stress. Markers such as the alpha asymmetry (AA) have been proposed to assess stress (Brouwer et al., 2011; Papousek et al., 2014). This marker is based on the difference in activity between left and right hemispheres. Despite the amount of EEG-based approaches, there is no established marker to assess stress by EEG.

In the present work, we propose an EEG-based marker for stress assessment: the prefrontal relative gamma power (RG). We focus on acute psychosocial stress (i.e., the type of stress induced by the MIST). This marker is based on the complementarity of fast and slow brain rhythms. It has been previously used in meditation-based studies (Lutz et al., 2004; Steinhubl et al., 2015), but not under pure relaxation/stress paradigms. Despite a direct relationship between meditation and relax states has not been demonstrated in the literature, it is usual in meditation studies to utilize relaxation/stress markers such as the HR (Kim et al., 2014; Steinhubl et al., 2015). In addition, results provided by this paper prove the usefulness of the prefrontal RG power for stress assessment. Among its advantages, the temporal resolution is higher than the one of other markers such as the HR or the cortisol. Moreover, it requires the use of few electrodes located at non-hairy head positions. These two features may result in the use of non-invasive dry wearable real-time devices for ubiquitous assessment of stress. These systems might help people to improve their life quality in diverse daily-life activities.

The paper is organized in four sections, including the present introduction (Section Introduction). Methods, subjects, and materials used during the study are reported in Section Methods. Afterwards, results obtained from data analysis are reported in Section Results. Finally, discussion of the results and conclusions are reported in section Discussion.

## Methods

### Experimental design

#### Subjects and data acquisition

Six healthy young volunteers (mean age, 26.3 ± 6.4 years) participated in the study. The subjects declared no previous experience in EEG or stress-related experiments. They were instructed not to take stimulants or relaxants during 24 h prior to the experiment. They wore hospital uniforms during the study. The protocol and informed consent were accepted by the Bioethics Committee of the University of Granada.

Once the informed consent was provided and signed by the subject, EEG, and electrocardiographic (ECG) signals were recorded at 540 Hz with the Miniature Data Acquisition System of Cognionics (Cognionics, Inc., USA). One ECG electrode was placed on the non-dominant wrist. Fifteen EEG electrodes were placed at Fp1, Fp2, Fz, F3, F4, F7, F8, Cz, C3, C4, Pz, T5, T6, O1, and O2 positions of the 10–20 International System. These positions have been included in reports of successful studies on emotions (Jenke et al., 2014). All the electrodes were referenced and grounded to the left ear lobe. The impedance of the electrodes was below 30 KΩ. This value is much lower than the input impedance of the acquisition system, and therefore signal degradation was insignificant.

#### Stress session

The subjects were stressed by the MIST (Dedovic et al., 2005). This procedure induces mental arithmetic load together with negative social feedback. It was demonstrated to increase levels of salivary free cortisol in healthy young people and was proposed as tool for functional imaging studies related to psychosocial stress. In fact, the MIST has been already used in various stress-related works (Dagher et al., 2009; Dedovic et al., 2009b; Nagano-Saito et al., 2013; Zschucke et al., 2015). In addition, a recent review included the MIST in the well-described methods to induce stress in humans (Bali and Jaggi, 2015).

The MIST consists of two stages namely, training and test. During the training stage, the subjects are asked to solve arithmetic operations without any time restriction. The arithmetic operations are organized in five difficulty levels and randomly displayed. During the test stage, the subjects must solve the same type of arithmetic operations with limited time. This limit is visually indicated to the subjects by a progress bar and calculated as the average time of correct answers in the training. The limit is adapted during the test stage depending on the number of consecutive wrong and right answers. In addition, the feedback for the current resolve (i.e., *correct, incorrect, timeout*) as well as the average performance are displayed after every single operation. The adaptive time limit enforces a range of about 20–45% performance whilst the subjects are asked to reach about 80–90% performance to be useful for the study. The subjects are periodically reminded of the importance of achieving the goal. This fact, together with the impossibility of reaching the asked performance, induces stress in subjects. See Dedovic et al. (2005) for a detailed explanation of the MIST.

**Figure 1:**
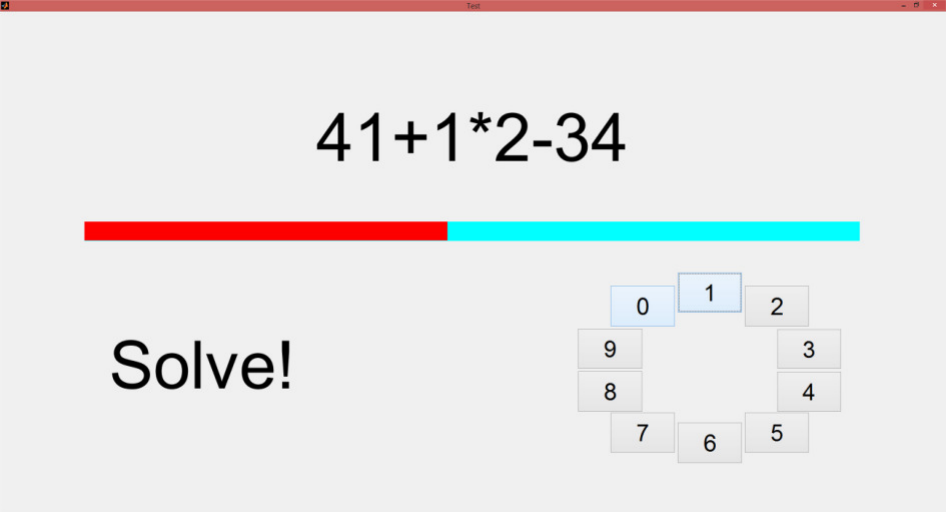
**The layout of the GUI implemented for the MIST**. On the top, the current calculation to be solved is displayed. In the middle, the red bar indicates the remaining time to solve the operation. At the bottom left, instructions and feedback (i.e., *correct, incorrect, timeout*) are displayed. At the bottom right, the button panel that provides the input. Solutions are ranged in the [0–9] interval.

In our study, the MIST was implemented using a Matlab (The MathWorks, Inc., USA) graphical user interface (GUI) running on a laptop (see Figure 1). The MIST was conducted within a classroom. The subjects were sitting on a comfortable chair. In order to avoid severe artifacts in EEG and ECG signals, they were instructed to exclusively move their hand using the touchpad (i.e., hand without the ECG electrode). The training stage and the test stage lasted, respectively, 3 and 6 min, following the indications in Dedovic et al. (2005). Therefore, the stress session lasted 9 min.

#### Relaxation session

A relaxation session was performed immediately after the stress session. The subjects stayed laid on a puff-shaped seat for 10 min, following the indications provided by a psychologist with a wide expertise in lighting-related treatments. The seat was placed inside a white-lighted closed room. The room was specially designed for relaxation. The subjects were instructed not to close their eyes (except for blinking), not to move, nor gaze any part of the room during the relaxation session. In order to check the behavior of the subjects, they were monitored by a video camera.

**Figure 2:**
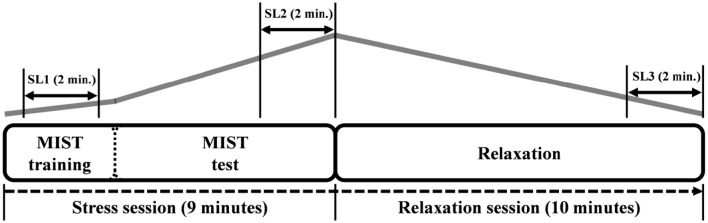
**The timeline of the experiment**. The first 3 min corresponds to the training part of the MIST. Afterwards, the MIST test is performed for 6 min. Then the relaxation session starts in the relaxation room and lasts 10 min. The gray line indicates the expected level of stress according to the paradigm. The three stress levels (SL1, SL2, and SL3) and the corresponding time periods are indicated over the gray line.

The timeline of the experiment and the expected stress level are displayed in Figure 2. Three stress levels were defined (i.e., SL1, SL2, and SL3). SL1 corresponds to the mean value during the 2 min in the middle of the MIST training. This period was chosen as initial stress level because the subjects generally started the training in a non-relaxed state due to several reasons (e.g., the stress produced by the EEG preparation and the instructions given by the technicians at the beginning of the experiment). SL2 corresponds to the mean value during the 2 last min of the MIST test. It should be the period of maximum stress level. Finally, SL3 corresponds to mean value during the 2 last min of the relaxation session. It should be the period of minimum stress level.

### Biosignals processing

#### EEG signals

Recorded EEG data were bandpass filtered using a second order Butterworth IIR filter with cutoff frequencies 1 and 100 Hz. A notch filter was applied to remove couplings from power-lines. Ocular artifacts were removed using independent component analysis.

After the preprocessing, a spectral analysis was performed. Two-second epochs (no overlap) were extracted, *z*-scored, and then the power spectral density (PSD) estimated for each EEG channel. The average power at different frequency bands was calculated through the area under the PSD in the intervals corresponding to the bands. These values were averaged across the channels to be jointly analyzed. The RG was computed as the power ratio between gamma (25–45 Hz) and slow rhythms (4–13 Hz). This spectral analysis is based on previous works using the RG (Lutz et al., 2004; Steinhubl et al., 2015). The absolute power at frequency bands theta (4–7 Hz), alpha (8–13 Hz), beta (14–24 Hz), and gamma (25–45 Hz) was also computed. For theta, alpha, and beta, it was the inverse value (i.e., 1/theta, 1/alpha, and 1/beta) for a better comparison with RG and HR. In addition, AA (i.e., relative difference in alpha power between left and right hemispheres) was calculated. This analysis was performed in different cortical areas such as prefrontal (Fp1, Fp2), frontal (Fz, F3, F4, F7, F8), central (Cz, C3, C4), and temporal-parietal (Pz, T5, T6). These frequency bands and cortical areas have been used in emotion-related works (Jenke et al., 2014).

All the results of the spectral analysis were smoothed with a moving average filter (30 samples) in order to better display them. In addition, in the group analysis (i.e., average across the six subjects), results were interpolated to fix inter-subject time warping, smoothed, *z*-scored, and then averaged. The averaged results were normalized by the maximum and the minimum (i.e., *y*_norm_ = [*y* − min(*y*)]/[max(*y*) − min(*y*)]).

#### ECG signals

Recorded ECG data were bandpass filtered using a second order Butterworth IIR filter with cutoff frequencies 4 and 24 Hz. This filter was applied in order to enhance the R peak of the QRS complex within the ECG signal (Semmlow, 2014). An automatic procedure for R peak detection was performed afterwards. Preprocessed ECG data were used to calculate the HR every 30 s by using a 90 s sliding window with 66% overlap factor.

In addition, in the group analysis (i.e., average across the six subjects), results were interpolated, *z*-scored and then averaged. The averaged results were normalized in a similar manner to EEG data (see Section EEG Signals).

### Statistical analysis

The mean of EEG power at different frequency bands and locations was computed over the time periods corresponding to SL1, SL2, and SL3. Mean of HR were also calculated over the same periods. The Wilcoxon signed-rank test was applied in order to assess whether mean ranks of repeated measurements (i.e., time periods of SL1, SL2, and SL3) significantly differ (*p* < α) with significance level α = 0.01. This test is usually used as an alternative to the paired Student's *t*-test when the distribution cannot be assumed to be normal (the Kolmogorov-Smirnov test was performed to check for normality). In addition, Pearson's linear correlation coefficient was computed to find correlations of EEG bands power and HR.

## Results

### EEG activity

**Figure 3:**
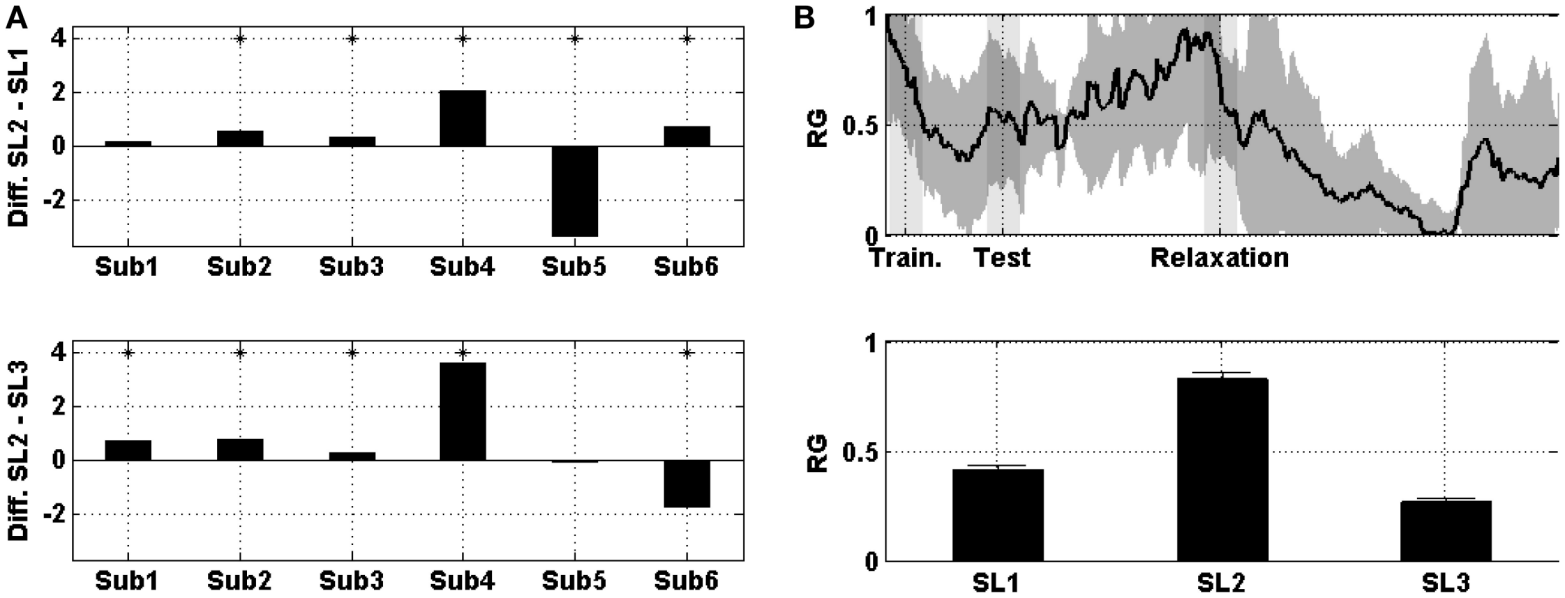
**(A)** On the top, the difference in the mean prefrontal RG between SL2 and SL1 across subjects. At the bottom, the difference between SL2 and SL3. Asterisks indicate statistically significant difference (Wilcoxon; *p* < 0.01). **(B)** On the top, the evolution of prefrontal RG averaged across the six subjects. Shades behind the curve indicate the standard error of the mean (SEM). Shaded bars indicate transition time intervals due to smoothing and interpolation. At the bottom, the mean value in SL1, SL2, and SL3. Horizontal bars indicate the SEM.

Figure 3A shows the difference in the mean prefrontal RG between SL1, SL2, and SL3. For subjects 2, 3, 4, 5, and 6 the difference between SL2 and SL1 was statistically significant (Wilcoxon; *p* < 0.01). For subject 5, the difference was negative (i.e., the RG was higher in SL1 than in SL2). Similarly, for subjects 1, 2, 3, 4, and 6 the difference between SL2 and SL3 was statistically significant (Wilcoxon; *p* < 0.01). For subject 6, the difference was negative (i.e., the RG was higher in SL3 than in SL2). Figure 3B shows the evolution of prefrontal RG averaged across the six subjects and then normalized. In the middle of the MIST training (i.e., SL1), the RG was below 0.5; at the end of the MIST test (i.e., SL2), the RG increased to up to 0.75 and, at the end of the relaxation session (i.e., SL3), the RG was around 0.25.

**Figure 4:**
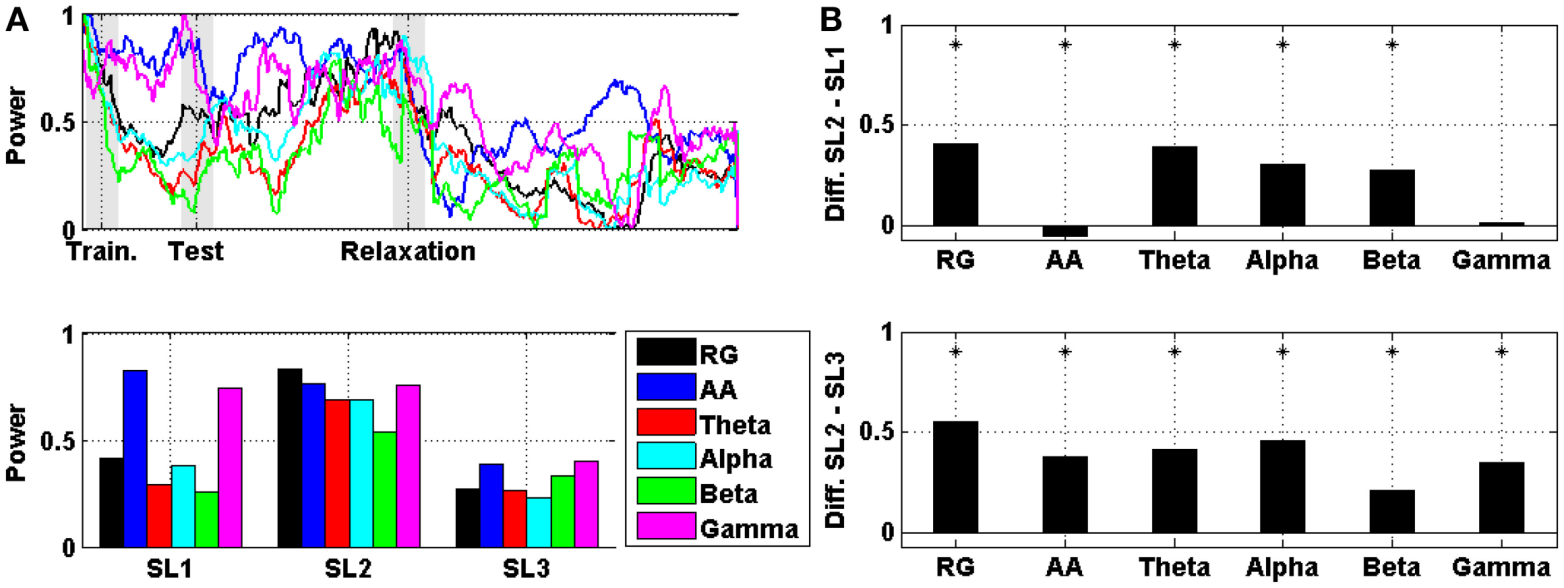
**(A)** On the top, the evolution of RG, AA, theta, alpha, beta, and gamma in prefrontal area averaged across the six subjects. Shaded bars indicate transition time intervals due to smoothing and interpolation. At the bottom, the mean value in SL1, SL2, and SL3. **(B)** On the top, the difference in the mean power between SL2 and SL1 for RG, AA, theta, alpha, beta, and gamma in prefrontal area. At the bottom, the difference between SL2 and SL3. Asterisks indicate statistically significant difference (Wilcoxon; *p* < 0.01).

A comparison between RG, AA, theta, alpha, beta, and gamma averaged across subjects (and then normalized) in prefrontal area is displayed in Figure 4. In particular, Figure 4A shows the evolution of the power, and Figure 4B shows the difference in the mean power between SL1, SL2, and SL3. For RG, AA, theta, alpha, and beta, the difference between SL2 and SL1 was statistically significant (Wilcoxon; *p* < 0.01). For AA, this difference was negative (i.e., the AA was higher in SL1 than in SL2). For RG, AA, theta, alpha, beta, and gamma, the difference between SL2 and SL3 was also statistically significant (Wilcoxon; *p* < 0.01). All these differences, together with those corresponding to other cortical areas (e.g., frontal, central, and temporal-parietal), are reported in Table 1. In all areas, the maximum difference between SL2 and SL3 was achieved using the RG (0.55, 0.57, 0.80, and 0.82 in prefrontal, frontal, central, and temporal-parietal areas, respectively). However, the maximum difference between SL2 and SL1 was achieved using the theta power in frontal (0.39), central (0.51) and temporal-parietal (0.47) areas, and using the RG in prefrontal area (0.41).

**Table 1:** **Differences in the mean power between SL1, SL2, and SL3 in the group analysis for RG, AA, theta, alpha, beta, and gamma in different cortical areas**.

	**Prefrontal**		**Frontal**		**Central**		**Temporal-parietal**	
	**SL2 – SL1**	**SL2 – SL3**	**SL2 – SL1**	**SL2 – SL3**	**SL2 – SL1**	**SL2 – SL3**	**SL2 – SL1**	**SL2 – SL3**
RG	0.41^*^	0.55^*^	0.36^*^	0.57^*^	0.36^*^	0.80^*^	0.33^*^	0.82^*^
AA	−0.06^*^	0.37^*^	0.05^*^	0.14^*^	−0.16^*^	−0.04^*^	−0.32^*^	−0.02
Theta	0.39^*^	0.42^*^	0.39^*^	0.53^*^	0.51^*^	0.61^*^	0.47^*^	0.66^*^
Alpha	0.31^*^	0.45^*^	0.35^*^	0.54^*^	0.30^*^	0.57^*^	0.35^*^	0.66^*^
Beta	0.28^*^	0.21^*^	0.25^*^	0.49^*^	0.13^*^	0.23^*^	0.26^*^	0.12^*^
Gamma	0.01	0.35^*^	0.12^*^	0.39^*^	0.20^*^	0.55^*^	0.11^*^	0.73^*^

Asterisks indicate statistically significant difference (Wilcoxon; p < 0.01). Shadings indicate maximum of each column.

Additionally, correlations of prefrontal RG with AA, theta, alpha, beta, and gamma in different areas are reported in Table 2. The highest correlations were RG with theta (0.89) and with alpha (0.89), both of them in frontal area. Theta power reached the maximum correlation in prefrontal (0.87) and frontal (0.89) areas. Alpha power was also in frontal area (0.89), and in central (0.87) and temporal-parietal (0.87) areas. Theta, alpha, and beta achieved their maximum correlation in frontal area (0.89, 0.89, and 0.82, respectively). On the other hand, AA and gamma had their maxima, respectively, in prefrontal (0.50) and central (0.82) areas.

**Table 2:** **Pearson's linear correlation coefficient and confidence interval (CI) for correlations in the group analysis of prefrontal RG with AA, theta, alpha, beta, and gamma in different cortical areas**.

	**Prefrontal**			**Frontal**			**Central**			**Temporal-parietal**		
	**CI low**	**Corr**	**CI up**	**CI low**	**Corr**	**CI up**	**CI low**	**Corr**	**CI up**	**CI low**	**Corr**	**CI up**
RG	1	1	1	0.96	0.97	0.97	0.88	0.90	0.91	0.82	0.84	0.86
AA	0.44	0.50	0.56	0.29	0.36	0.42	−0.32	−0.24	−0.17	0.03	0.11	0.19
Theta	0.85	0.87	0.89	0.87	0.89	0.91	0.76	0.80	0.82	0.80	0.83	0.85
Alpha	0.84	0.86	0.88	0.87	0.89	0.91	0.85	0.87	0.89	0.85	0.87	0.89
Beta	0.52	0.57	0.62	0.79	0.82	0.85	0.62	0.67	0.71	0.40	0.47	0.53
Gamma	0.78	0.81	0.84	0.76	0.79	0.82	0.80	0.82	0.85	0.74	0.77	0.80

Shadings indicate maximum for each cortical area.

### ECG activity

**Figure 5:**
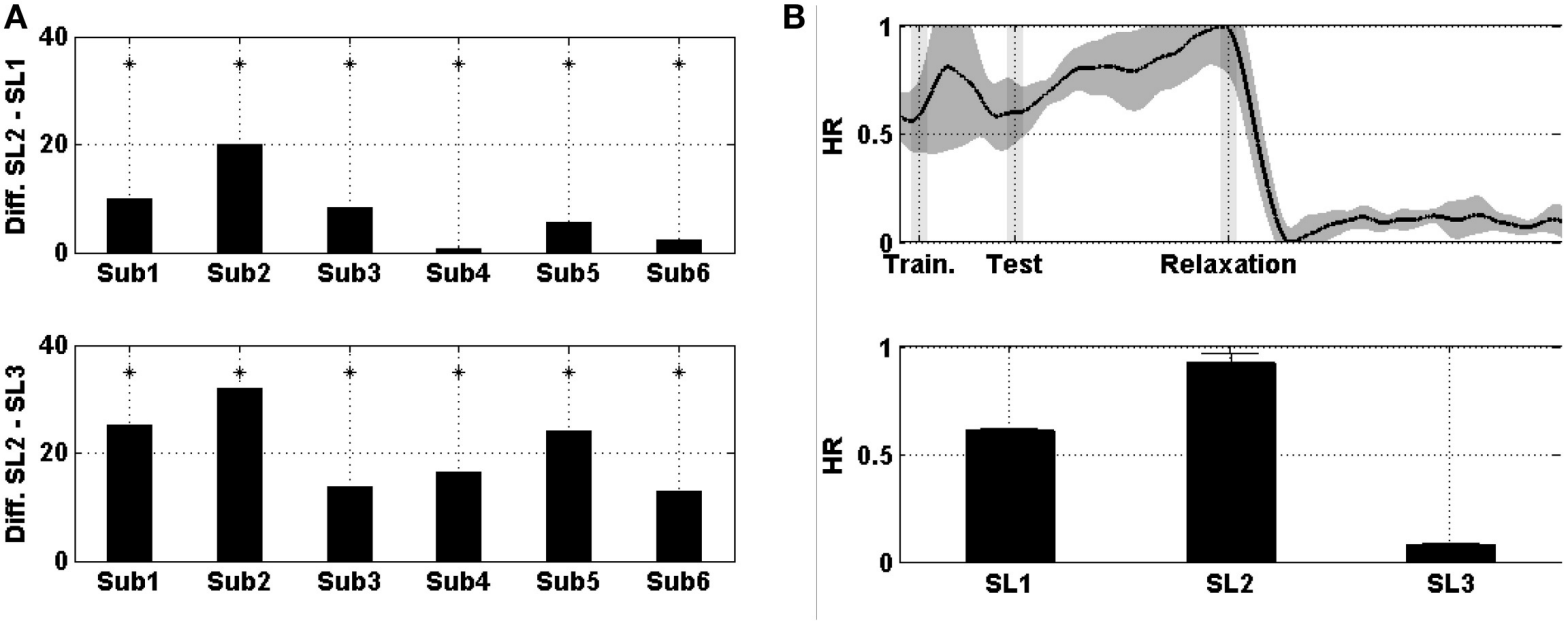
**(A)** On the top, the difference in the mean HR between SL2 and SL1 across subjects. At the bottom, the difference between SL2 and SL3. Asterisks indicate statistically significant difference (Wilcoxon; *p* < 0.01). **(B)** On the top, the evolution of the HR averaged across the six subjects. Shades behind the curve indicate the SEM. Shaded bars indicate transition time intervals due to smoothing and interpolation. At the bottom, the mean value in SL1, SL2, and SL3. Horizontal bars indicate the SEM.

Figure 5A shows the difference in the HR between SL1, SL2, and SL3. For every single subject, the differences between SL2 and SL1, as well as between SL2 and SL3, were statistically significant (Wilcoxon; *p* < 0.01). Figure 5B shows the evolution of the HR averaged across the six subjects and then normalized. In the middle of the MIST training (i.e., SL1), the HR was a little above 0.6; at the end of the MIST test (i.e., SL2), the HR increased to up to around 0.9 and, at the end of the relaxation session (i.e., SL3), the HR was around 0.1.

**Figure 6:**
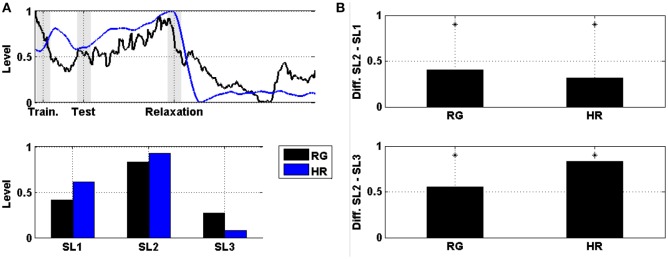
**(A)** On the top, the evolution of the level of prefrontal RG together with the HR averaged across the six subjects and then normalized. Shaded bars indicate transition time intervals due to smoothing and interpolation. At the bottom, the mean value in SL1, SL2, and SL3. **(B)** On the top, the difference in the mean power between SL2 and SL1 for the level of prefrontal RG and HR. At the bottom, the difference between SL2 and SL3. Asterisks indicate statistically significant difference (Wilcoxon; *p* < 0.01).

The comparison between levels of prefrontal RG and HR averaged across subjects and then normalized is displayed in Figure 6. Figure 6A shows the evolution of these levels, and Figure 6B shows the difference in the mean power between SL1, SL2, and SL3. For both markers (i.e., prefrontal RG and HR), the difference between SL2 and SL1, as well as between SL2 and SL3, were statistically significant (Wilcoxon; *p* < 0.01). These values are reported in Table 3. The maximum difference between SL2 and SL1 was achieved using the prefrontal RG (0.41). On the contrary, the maximum difference between SL2 and SL3 was reached by the HR (0.84).

**Table 3:** **Differences in the mean level of prefrontal RG and HR between SL1, SL2, and SL3 in the group analysis**.

	**SL2 – SL1**	**SL2 – SL3**
Prefrontal RG	0.41^*^	0.55^*^
HR	0.31^*^	0.84^*^

Asterisks indicate statistically significant difference (p < 0.01). Shadings indicate maximum of each column.

Finally, correlations of HR with RG, AA, theta, alpha, beta, and gamma in different cortical areas are reported in Table 4. The highest correlation was RG in central area (0.95). Alpha power reached the maximum correlation in prefrontal (0.81) and frontal (0.86) areas. However, RG was in central (0.95) and temporal-parietal (0.94) areas. Theta and alpha achieved their maximum correlation in temporal-parietal area (0.88 and 0.92, respectively). Beta had its maximum in frontal area (0.85). Gamma and RG achieved their maxima in central area (0.93 and 0.95, respectively). The AA reached its maximum in prefrontal area (0.75).

**Table 4:** **Pearson's linear correlation coefficient and confidence interval (CI) for correlations in the group analysis of HR with RG, AA, theta, alpha, beta, and gamma in different cortical areas**.

	**Prefrontal**			**Frontal**			**Central**			**Temporal-parietal**		
	**CI low**	**Corr**	**CI up**	**CI low**	**Corr**	**CI up**	**CI low**	**Corr**	**CI up**	**CI low**	**Corr**	**CI up**
RG	0.77	0.80	0.83	0.83	0.85	0.87	0.94	0.95	0.95	0.93	0.94	0.95
AA	0.71	0.75	0.78	0.30	0.37	0.43	−0.17	−0.09	−0.01	0.28	0.35	0.42
Theta	0.59	0.64	0.68	0.72	0.76	0.79	0.72	0.75	0.78	0.86	0.88	0.89
Alpha	0.79	0.81	0.84	0.83	0.86	0.88	0.88	0.90	0.91	0.91	0.92	0.93
Beta	0.42	0.48	0.54	0.82	0.85	0.87	0.54	0.60	0.65	0.33	0.40	0.46
Gamma	0.70	0.74	0.77	0.75	0.79	0.81	0.92	0.93	0.94	0.92	0.93	0.94

Shadings indicate maximum for each cortical area.

## Discussion

In this work, the RG was used to assess changes in stress level of healthy subjects. To the best of our knowledge, RG has been previously used to assess meditation states with expert and novice meditators (Lutz et al., 2004; Steinhubl et al., 2015). The RG has been never utilized as a marker to quantify stress level during relaxation/stress sessions. The cited meditation-related works found contrary results regarding the positive or negative correlation between the RG and the meditation level. Our results showed a positive correlation of the RG with the stress level, in particular, with the expected stress level (see Figure 2) and with the HR (0.8).

The prefrontal RG was able to significantly differentiate for 5 out of 6 subjects in case of SL1 to SL2, and for the 6 subjects in case of SL2 to SL3. Nevertheless, these differences had negative sign for a couple of subjects, thus indicating a reverse behavior (i.e., not expected) in these cases. The group analysis showed that the RG was the most discriminative marker in prefrontal area for both SL transitions. It is the same for the transition 2–3 in all other areas. However, for the transition 1–2, theta power was the most discriminative marker in frontal, central, and temporal-parietal areas. This fact could have been caused by changes in task attention. Although the AA has been utilized in various recent stress-related works (Brouwer et al., 2011; Papousek et al., 2014), in the present study, the RG was more discriminative than the AA for both SL transitions in all the cortical areas, and therefore better stress marker. This outcome suggests the alternative use of the RG to assess stress level. Results reported in Table 1 showed that there generally were significant differences in EEG bands power between stress levels in every single area. However, according to related literature, stress is reflected by changes in regions of prefrontal and frontal areas such as the orbitofrontal regions, frontal lobes, and medium prefrontal cortex (Tanida et al., 2007; Dedovic et al., 2009a,b; Nagano-Saito et al., 2013; Papousek et al., 2014). Focusing on those areas, theta and alpha waves were the most weighted components of the RG since gamma waves did not significantly change from SL1 to SL2. It suggests that prefrontal gamma is related to cognitive processes (which remain in both stress levels) rather than psychosocial stress. In fact, this was claimed in previous literature (Başar-Eroglu et al., 1996). It may be important to consider both gamma and slow rhythms (i.e., theta + alpha) in order to assess full stress level, including cognitive and psychosocial relax. Nevertheless, in central area, gamma power did significantly increase for transition 1–2. Indeed, a recent study concluded that high frequency cortical activity measured through Cz electrode was related to affective processing (Sirca et al., 2015), which could be related to stress.

Regarding the HR and its comparative with the EEG, the MIST increased the HR of the subjects and the relaxation session decreased it. This was expected since the HR has been proved to be related with the stress level in previous works (Sayette, 1993; Chandiramani et al., 2007; Ranganathan et al., 2012; Reinhardt et al., 2012; Michels et al., 2013; Regula et al., 2014; Dimitriev and Saperova, 2015). The RG highly correlated with the HR (almost 0.8). However, it was not the maximum correlation in frontal and prefrontal areas. In addition, the maximum correlation of RG with HR was achieved in central cortex. This suggests that prefrontal activity can reflect little changes in stress level that cannot be indicated by neither central EEG nor HR. As mentioned, the maximum difference between SL2 and SL3 was achieved using the HR, but it was the prefrontal RG between SL2 and SL1. It may be due to there was a HR peak during the 2 min of SL1. In general, the HR was more discriminative than the prefrontal RG according to the subject-by-subject and the group analysis. However, the prefrontal RG has better temporal resolution. This advantage might be essential in potential development of real-time devices for online assessment of stress. In addition, the fact of getting reliable measures of stress through few prefrontal electrodes (Fp1, Fp2) facilitates the use of wearable devices for that proposal.

We are aware of the inherent difficulties to follow a sound methodology when EEG is involved in non-controlled experiments (i.e., out-of-the-lab experiments with motor and cognitive artifacts). The conducted experiment was designed to overcome these limitations with a clear and reproducible methodology. Since participants could start the experiment with unknown levels of stress, it was necessary to expose them to a condition in which a homogenous level of stress was caused before applying stimulation. If participants had started the experiment from “zero stress level,” no relaxing effects could have observed in any case. A well-established method (i.e., the MIST) was used for that. Stress was undoubtedly caused. In addition, the HR marker also indicated increasing levels of stress during the MIST (see Figure 5). We understand that this fact is not under discussion. The way in which the prefrontal RG was successfully measured during the MIST indicates the robustness of our EEG experiment under these adverse circumstances. Right after the stress session, participants got into an isolated room and EEG was recorded under conditions close to the ones of typical EEG experiments. The participants got relaxed after 10 min. laying on a puff-shaped seat in our specific room designed to cause relax (broadly used in Education Centers after an outbreak of violence to cause relax), isolated of disturbances and with no stimulation apart of the white lighting. As expected, the HR marker indicated decreasing levels of stress during the relaxation session (see Figure 5). For the authors, it is unquestionable that the participants got relaxed at the end of the relaxation session. Under both circumstances, stress and relaxation, we found the main claim of this paper: a correlation of the prefrontal RG with the stress level.

In conclusion, we found that the prefrontal RG can be used as a marker for stress assessment. It has been previously used in meditation studies, but not under relaxation/stress paradigms. We analyzed and compared it with several EEG frequency bands and with the HR during relaxation/stress sessions. The prefrontal RG significantly discriminated stress levels, and highly correlated with the expected stress level and the HR. The paper reports the methodology and results of a preliminary study that can motivate further research in the field. Only six subjects participated in the study. In addition, it is difficult to determine a “healthy volunteer” about stress since there are many constraints (e.g., social, family, personal, and work) that may influence stress as to restrict the study to only six people. Therefore, more case studies are needed to draw more accurate and reliable conclusions. Despite that, our findings could have relevant impact on stress assessment research. The assessment of stress level by the prefrontal RG has two main advantages. On one hand, the prefrontal RG has higher temporal resolution than other established stress markers such as the HR or the cortisol. On the other hand, it implies the use of few electrodes located at non-hairy head positions. Therefore, it facilitates the use of non-invasive dry wearable real-time devices for ubiquitous assessment of stress, thus potentially helping to improve the life quality of people in daily-life activities.

## Author contributions

JM is the main contributor of this work. He participated in the design of the experimental protocol, conducted the study, analyzed the data, discussed the results, and wrote the paper. ML and FP participated in the design of the experimental protocol, provided guidelines for the development of the study and the data analysis, discussed the results and revised the paper.

## Funding

This work was supported by Nicolo Association for the R+D in Neurotechnologies for disability, the Ministry of Economy and Competitiveness DPI2015-69098-REDT, the research project P11-TIC-7983 of Junta of Andalucia (Spain), and the Spanish National Grant TIN2015-67020-P, co-financed by the European Regional Development Fund (ERDF).

### Conflict of interest statement

The authors declare that the research was conducted in the absence of any commercial or financial relationships that could be construed as a potential conflict of interest.

## Footnotes

^2^www.stress.org
